# Phenotypic and Molecular Characterization of K54-ST29 Hypervirulent *Klebsiella pneumoniae* Causing Multi-System Infection in a Patient With Diabetes

**DOI:** 10.3389/fmicb.2022.872140

**Published:** 2022-05-31

**Authors:** Chunhong Shao, Li Xin, Peiyan Mi, Meijie Jiang, Haiyan Wu

**Affiliations:** ^1^Department of Clinical Laboratory of Shandong Provincial Hospital Affiliated to Shandong First Medical University, Jinan, China; ^2^Cardiac Department of Taian City Central Hospital, Tai’an, China; ^3^Intensive Care Department of Taian City Central Hospital, Tai’an, China; ^4^Clinical Laboratory of Taian City Central Hospital, Tai’an, China

**Keywords:** *Klebsiella pneumoniae*, hypervirulent, K54, diabetes, China

## Abstract

Worldwide, hypervirulent *Klebsiella pneumoniae* (hvKp) is one of the leading causes of multisystem infection. Serotype K54 has also been considered as one of the hvKp-associated capsular types that are rarely reported. In this study, we reported a K54-ST29 hvKp isolated from a 58-year-old male patient with diabetes in a teaching hospital in China. The patient rapidly developed sepsis and brain abscess, with a lethal multiple-organ-system failure due to K54 hvKp infection. This K54 hvKp isolate showed high level of toxicity in a mouse infection model and was susceptible to all the tested antibiotics. The isolate was fully sequenced, and its genome was compared with the available K54 *K. pneumoniae* genome. We predicted 133 virulence and pathogen-related genes, including those involved in fimbriae synthesis, iron transport, and enterobactin synthesis. Sequence alignment revealed >90% similarity among seven K54 *K. pneumoniae* strains. Our data suggest that community-acquired infection caused by hypervirulent K54 *K. pneumoniae* in patients with diabetes is a concern in East Asia.

## Introduction

*Klebsiella pneumoniae* (*K. pneumoniae*) is a common pathogen causing various nosocomial infections, including pneumonia, urinary tract infection, abdominal infection, and bacteremia. *K. pneumoniae* is now recognized as an urgent threat to human health, because of the emergence of multidrug-resistant strains associated with hospital outbreaks and hypervirulent strains associated with severe community-acquired infections (Shao et al., 2021a). A unique case of *K. pneumoniae* infection leading to liver abscess along with endophthalmitis was reported for the first time in Taiwan in the 1980s, and the causative organism was designated as hypervirulent *K. pneumoniae* (hvKp). Hence, hvKp has been recognized as another pathotype in addition to classical *K. pneumoniae* (cKp), associated with high degree of pathogenicity and mortality due to hypervirulence (Zhu et al., 2021). Currently, hvKp isolates have been reported mainly in Asia, Europe, and North America, and more recently in South America (Morales-León et al., 2021).

After invasive *K. pneumoniae* infections became a recognized clinical concern in Asia, several studies on the isolates reported the differences between cKp and hvKp. However, there is no specific feature that captures all hvKp strains to date (Russo and Marr, 2019). Clinical manifestations, capsule typing, the hypermucoviscous phenotype, and presence of virulence associated genes could be used to differentiate hvKp from cKp strains (Choby et al., 2020). The capsule polysaccharide of *K. pneumoniae* has been viewed as an important virulence factor that promotes resistance to phagocytosis and serum bactericidal activity. Currently, more than 100 capsular (K) serotypes of *K. pneumoniae* have been identified. Notably, many reports have shown that K1 and K2 serotypes are strongly associated with hvKp (Wyres et al., 2016). Nevertheless, capsular type K54, particularly representatives of sequence type (ST) 29, has also been associated with hypervirulence (Turton et al., 2018). In this study, we isolated a K54-ST29 *K. pneumoniae* from a patient with diabetes suffering multiple system infection in a Chinese Hospital. We characterized the genotypic and phenotypic features of the isolate.

## Materials and Methods

### Bacterial Strains

Three strains of *K. pneumoniae* were isolated from two blood cultures and a liver abscess puncture fluid of a 58-year-old man admitted to endocrine and immunology ward of a teaching hospital in Shandong Province of China on February 9, 2020. Four days prior to admission, the patient had experienced fatigue, anorexia, fever, and chills, without temperature monitoring and special treatment. He had a history of type 2 diabetes (> 20 years). The patient had no history of traveling abroad. Signed informed consent was obtained from the patient involved in this study. Patient information was obtained from electronic medical records. The methods in this study were approved by the Ethics Committee of Taian City Central Hospital (No. 2021-05-30), and the study was conducted in accordance with the approved guidelines. The isolates were identified as *K. pneumoniae* using VITEK-2 compact system and confirmed using VITEK-MS system (BioMérieux, France). Two strains of *K. pneumoniae* isolated from blood samples were designated as TAKPN-1 and TAKPN-3, and the strain isolated from liver abscess was named TAKPN-2.

### Determination of Hypermucoviscosity Phenotype and Serotype Analysis

The hypermucoviscosity phenotype was determined by the string test. Samples were cultured on blood agar plates overnight at 37°C. A colony from the plate was then streaked with a bacteriology loop. A viscous string over 5 mm in length was considered as a positive result. As described previously, the isolate was serotyped for K1, K2, K5, K20, K54, and K57 serotypes (Shao et al., 2021b).

### Antibiotic Susceptibility Assay

Antimicrobial susceptibility testing was performed using VITEK-2 compact system (BioMérieux, France). The minimum inhibitory concentrations (MICs) of imipenem, meropenem, and ertapenem were determined through an E-test (BioMérieux, France). *Escherichia coli* ATCC25922 and *K. pneumoniae* ATCC700603 served as the quality controls. All antibiotics were administered according to the approved standard of the 2021 European Committee on Antimicrobial Susceptibility Testing breakpoint.^1^

### Mouse Lethality Assay

To determine the virulence of three *K. pneumoniae* isolates, pathogen-free, 6–8 week-old, female C57BL/6 mice [Changzhou Cavion Experimental Animal Co, Ltd. (license number SCXY (Su) 2011-0003)] were used. In the present study, 10 mice were used as a sample population for each bacterial concentration. *Klebsiella pneumoniae* ATCC 13883 served as the control. C57BL/6 mice were infected intraperitoneally with 0.1 ml of bacterial suspension at a concentration of 10^5^ CFU in 0.9% NaCL. Symptoms and mortality rates were observed for 10 days. Further, we obtained the liver, lung, brain, and blood of dead mice for bacterial culture.

### Multilocus Sequence Typing

Multilocus sequence typing (MLST) of *K. pneumoniae* was performed according to protocols available on the MLST Pasteur website.^2^ Seven conserved housekeeping genes (*gapA*, *infB*, *mdh*, *pgi*, *phoE*, *rpoB*, and *tonB*) were amplified, sequenced, and compared with those in the MLST databases.

### Pulse-Field Gel Electrophoresis

An overnight grown bacterial culture in LB medium at 37°C was centrifuged and suspended in cell suspension buffer [100 mM EDTA, 100 mM Tris–HCl (pH 8.0)] and adjusted to an optical density (OD) of 4.0 at a wavelength of 600 nm. The suspension was mixed with equal volumes 2% solution of low melting agarose in Tris-EDTA [TE: 1 mM EDTA, 10 mM Tris–HCl (pH 8.0)]. After cooling, the agarose sections were incubated for 4 h at 54°C in cell lysis buffer [50 mM Tris–HCl, 50 mM EDTA (pH 8.0), 0.01 g/ml N-lauroyl-sarcosine, sodium salt, and 0.1 mg/ml proteinase K]. Thereafter, the sections were washed thoroughly with TE buffer and digested overnight with XbaI restriction endonuclease (Takara Bio, Inc., Otsu, Japan). Genomic DNA was separated in 0.5 × Tris/borate/EDTA (TBE) buffer in a Pulse-Field Gel Electrophoresis (PFGE) system (CHEF Mapper; Bio-Rad Laboratories, Inc., Hercules, CA, United States) at 14°C, using a voltage of 6 V/cm, a switch angle of 120°, and a switch ramp of 6–36 s for 21 h.

### Genomic Analysis

The draft genome sequence of TAKPN-1 was determined by the Shanghai OE Biotech Co., Ltd. (Shanghai, China). Sequencing was performed on an Illumina HiSeq Xten platform (Illumina Inc., San Diego, CA, United States). An Illumina shotgun library using the Illumina TruSeq Nano DNA LT Sample Prep Kit was reconstructed and sequenced in paired ends using the Illumina HiSeq platform. Raw sequencing data were generated using the Illumina base-calling software CASAVA v1.8.23, according to the manufacturer’s protocol. The sequenced reads were assembled using SOAPdenovo software (Feng et al., 2021).

Contigs with length greater than 500 bp were annotated by the NCBI prokaryotic genome annotation pipeline (PGAP) and Rapid Annotation using Subsystem Technology web server (RAST; http://rast.nmpdr.org/). The genomic sequences were additionally annotated with KEGG databases^3^ to analyze the metabolic pathway. Bacterial virulence factors (VFDB) and pathogen host interaction (PHI) factors were further predicted by Virulence Factors of Pathogenic Bacteria database^4^ and PHIs Database^5^ respectively, considering *a* ≥ 80% similarity criteria. Additionally, the genome of K1 hvKp (NTUH-K2044), K2 hvKp (SMU18037509), and cKp (ATCC 13883) was retrieved from National Center for Biotechnology Information (NCBI) database. Bacterial virulence factors of TAKPN-1 were compared with that of NTUH-K2044, SMU18037509, and ATCC13883.

### Phylogenetic Tree Construction

The genome of *K. pneumoniae* with Capsular Genotype K54 was retrieved from NCBI database for the query “K54 and *K. pneumoniae*.” We obtained eight genome of K54 *K. pneumoniae*. The maximum likelihood method was used to construct the phylogenetic tree, and the software used was PhyML v3.0^6^ with bootstrap as 1,000.

## Results

### Clinical Characteristics of Patient

**Figure 1:**
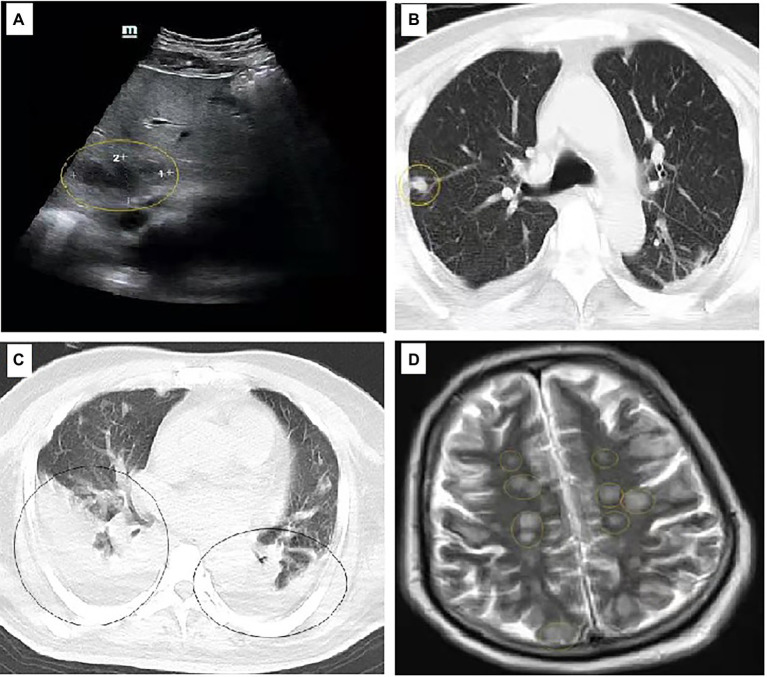
Image findings in this case. **(A)** Abdominal ultrasound showed the presence of intrahepatic abscess; **(B,C)** An computed tomography (CT) scan revealed an abscess of right lung and bilateral pleural effusion. **(D)** The nuclear MRI showed multiple brain abscess. Yellow circles refer to the abscesses and black circles refer to the presence of pleural effusion.

The patient was admitted with fever (39.5°C) and normal blood pressure. Laboratory exams showed increased leukocytosis with neutrophilia (18.92 × 10^6^ cells per liter with 88.5% neutrophils) and procalcitonin (7.21 mg/L). Abdominal ultrasound detected intrahepatic abscess (Figure 1A). A computed tomography (CT) scan revealed an abscess of right lung and bilateral pleural effusion (Figures 1B,C). Moreover, the nuclear MRI showed multiple brain abscess (Figure 1D). On February 15, 2020, two strains of *K. pneumoniae* were isolated from blood cultures and a liver abscess puncture fluid. On February 20, 2020, lumbar puncture revealed turbid yellow cerebrospinal fluid (CSF) with a cell count of 40,500 cells/μl (normal range, <5 cells/μl). On February 22, 2020, we obtained the bronchoalveolar lavage fluid (BALF) of the patient and conducted culture and gene analysis of pathogenic microorganisms. Unfortunately, we were not successful in obtaining pathogenic bacteria from CSF and BALF. However, gene analysis of BALF revealed the presence of *K. pneumoniae*.

**Figure 2:**
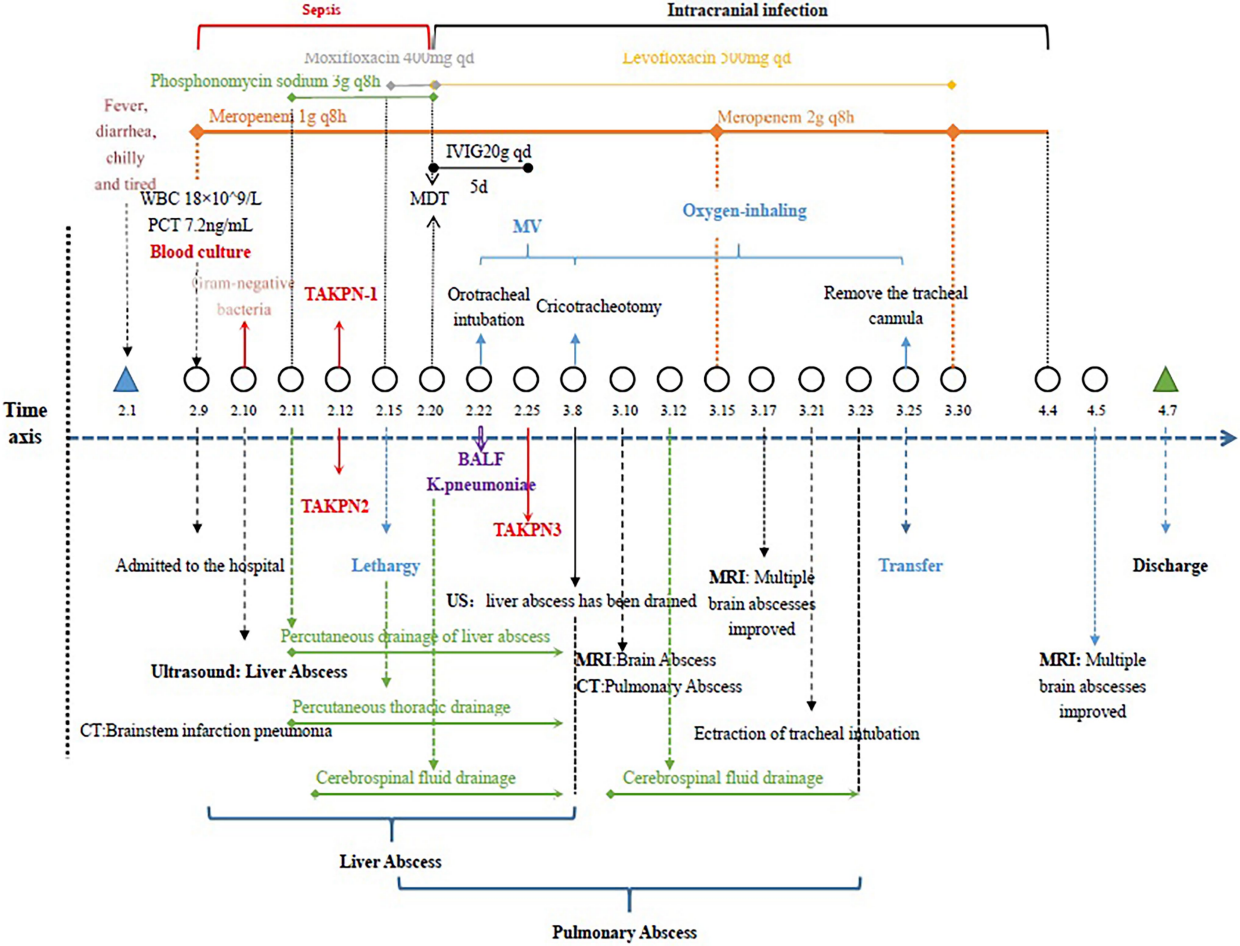
Clinical characteristics and treatment process of the patient.

The patient was treated with intravenous meropenem (1 or 2 g/8 h) from February 9, 2020, to April 4, 2020, and fosfomycin, moxifloxacin, and levofloxacin were used in intermittent combination during this period. During hospitalization, lumbar puncture and ventilator assisted respiration were performed. The specific treatment scheme and process are shown in Figure 2. The patient was discharged on April 7, 2020.

### Drug Sensitivity Results and MLST and PFGE of Three *Klebsiella pneumoniae* Isolates

Three *K. pneumoniae* isolates were susceptible to all tested antibiotics, including aztreonam, ampicillin/sulbactam, piperacillin/tazobactam, cephalosporins, carbapenems, quinolones, trimethoprim/sulfamethoxazole, and aminoglycosides (Supplementary Table S1). MLST revealed that the sequence type of three *K. pneumoniae* isolates was ST29. PFGE showed that three *K. pneumoniae* isolated from different parts showed 100% genetic similarity (Supplementary Figure S1).

### Virulence of Three *Klebsiella pneumoniae* Isolates

**Figure 3:**
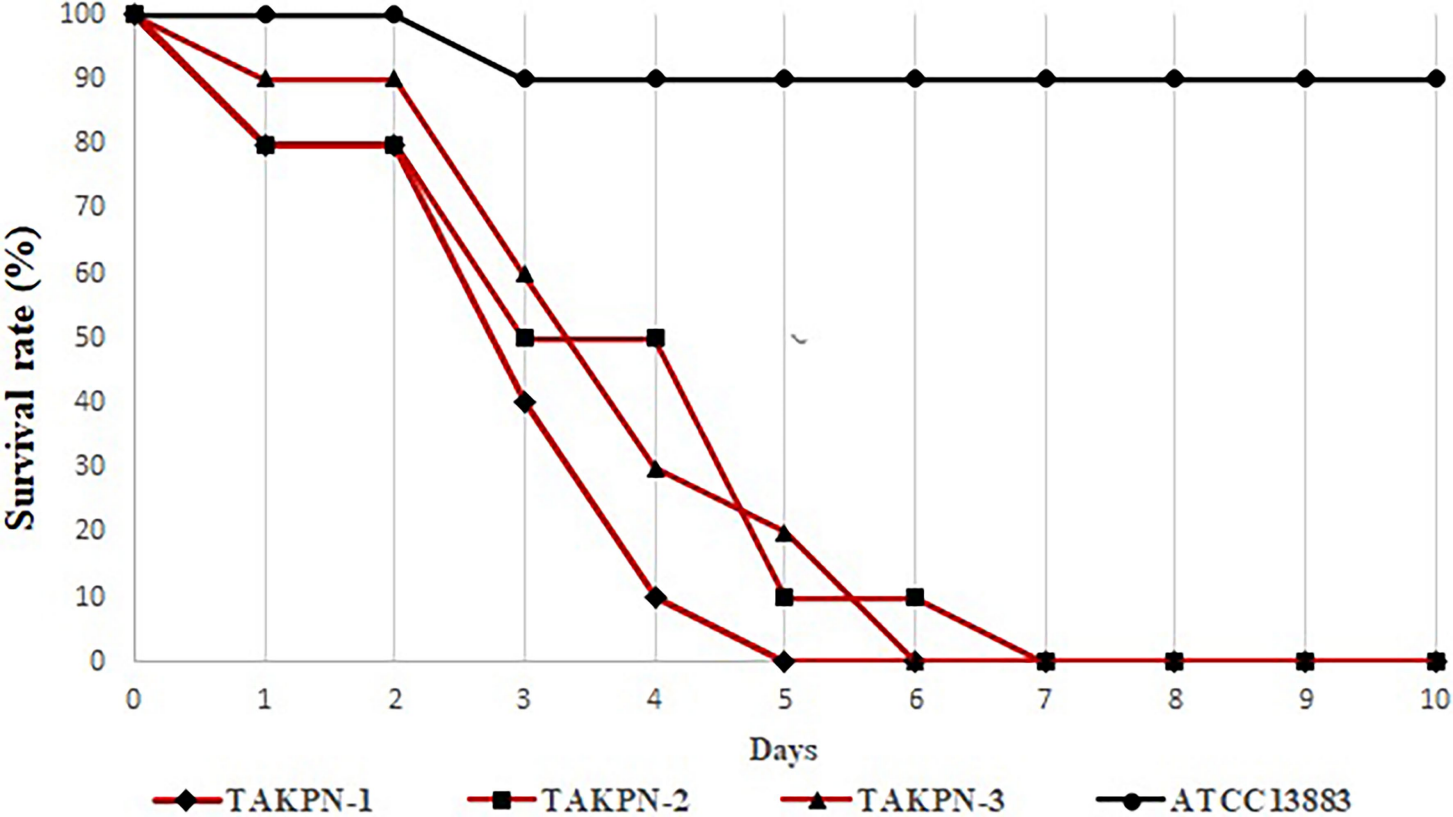
The survival rate of mouse infected by three hypervirulent *Klebsiella pneumoniae* (hvKP) isolates. *Klebsiella pneumoniae* ATCC 13883 served as the control.

The string test of three isolates showed positive results. PCR amplification determined their K54 serotype. In the mouse infection experiment, the three *K. pneumoniae* isolates showed similar higher toxicity compared with *K. pneumoniae* ATCC 13883. The mean mortality of mice on the third and fifth days was 50 and 90%, respectively. On the seventh day, all mice infected with clinical isolates died. However, the mortality of mice infected with *K. pneumoniae* ATCC 13883 was only 10% (Figure 3). Furthermore, *K. pneumoniae* were successfully obtained from the blood, lung, liver, and brain tissue of mice infected by TAKPN-1, TAKPN-2, and TAKPN-3. However, no bacteria were obtained from the tissues and blood of mice infected with *K. pneumoniae* ATCC 13883.

### Genomic Characterization of TAKPN-1

The draft genome size of the TAKPN-1 was determined to be 5,127,042 bp and the GC content was calculated to be 57.64%. A total of 4,714 predicted genes were annotated of which 4,477 were protein-coding genes and 237 were RNA genes (25 rRNAs, 85 tRNAs, and 127 sRNAs). KEGG analyses detected 2,638 genes involved in 22 biological functions in the draft genome of the TAKPN-1. Moreover, TAKPN-1 carried a limited number of antibiotic resistance genes. This also corresponded to the phenotypic results of antibiotics sensitivity test.

**Figure 4:**
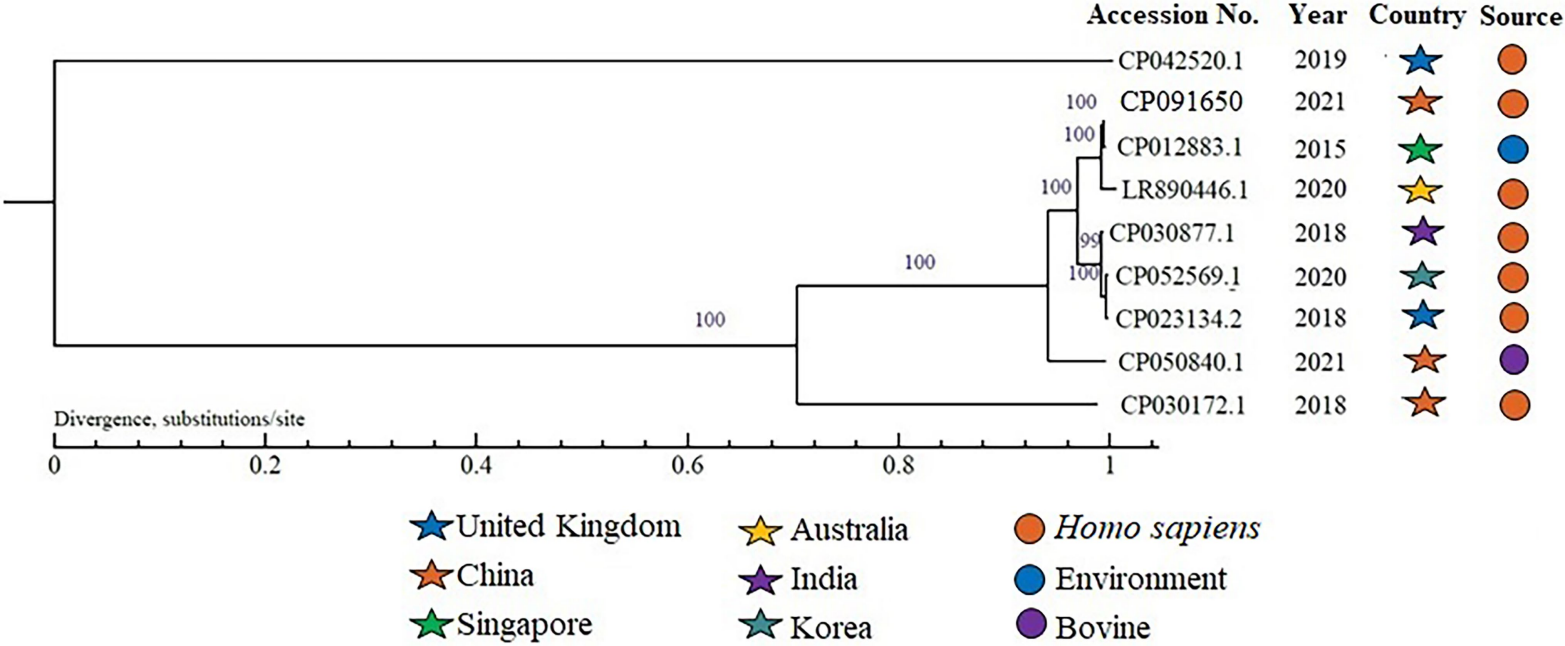
A phylogenetic tree of nine K54-hvKP isolates. The numbers on the branch indicate the branch reliability. The closer the value is to 100, the higher the reliability is. The branch length represents the evolutionary distance, which is calculated by the average number of substitutions per nucleotide.

The complete sequence of TAKPN-1 was submitted to GenBank under accession number CP091650. The full published sequences of eight *K. pneumoniae* of K54 were downloaded and compared with that of TAKPN-1. We found that three out of nine isolates were from China, and seven out of nine strains were isolated from Homo sapiens. Sequence alignments revealed more than 90% similarity among the seven isolates, excluding CP042520.1 and CP030172.1 (Figure 4).

### Identification of Virulent Factors and Pathogen Host Interaction Factors

**Figure 5:**
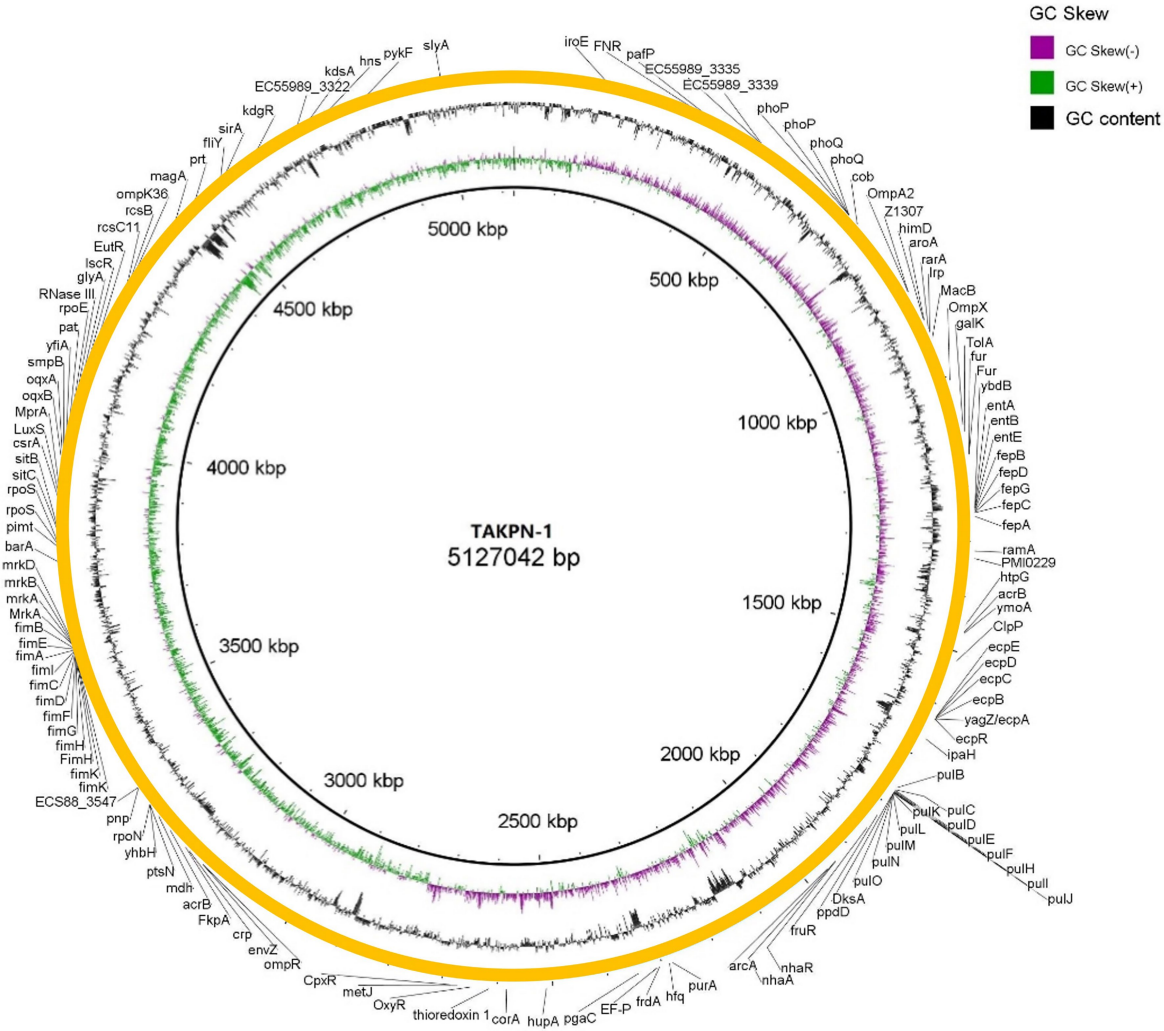
The genome of isolate TAKPN-1. Bacterial virulence factors and pathogen host interaction (PHI) factors with identity ≥80% were depicted. The identity represents the sequence similarity between the genes of TAKPN-1 and those of the reference genome.

**Figure 6:**
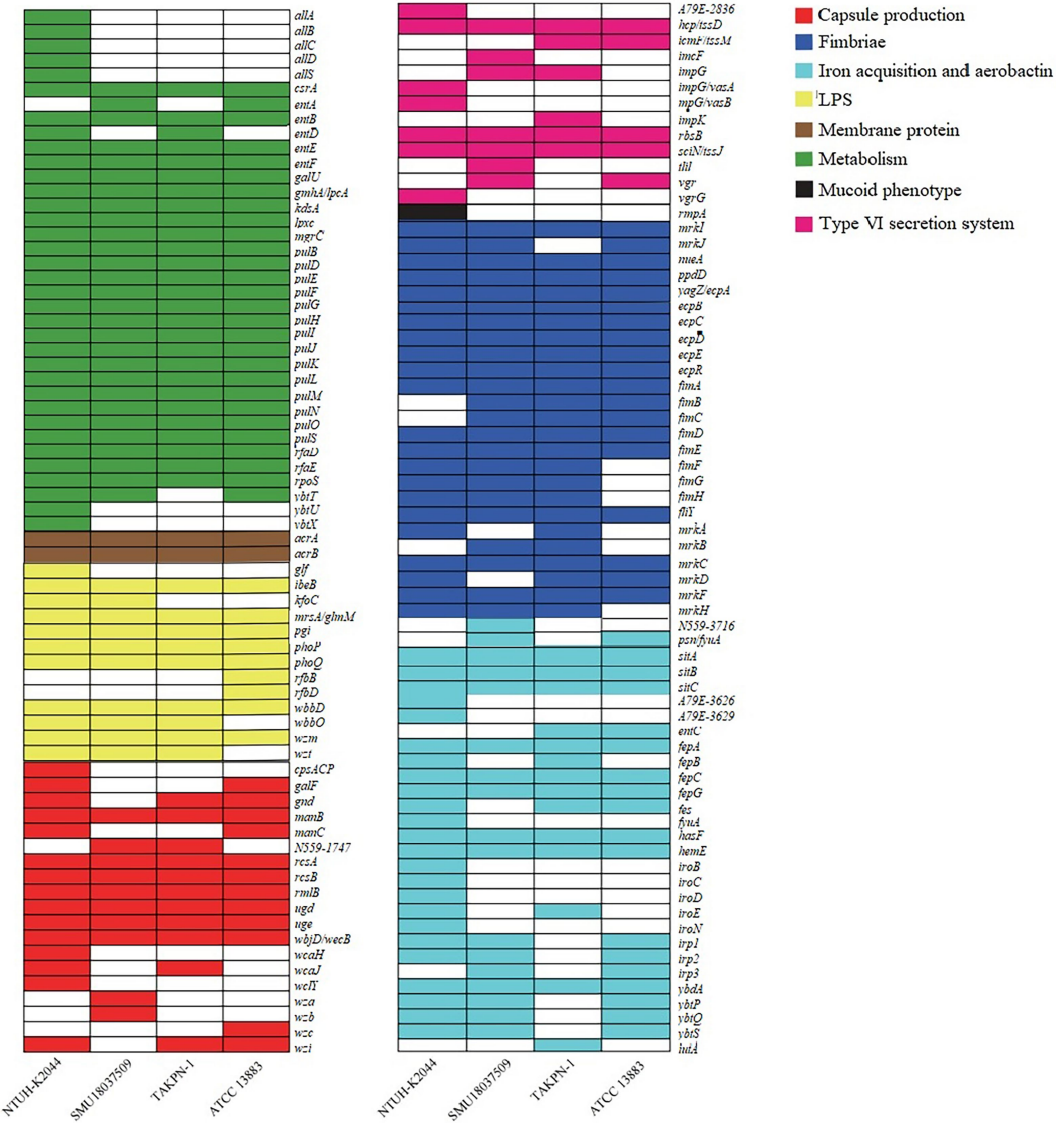
Bacterial virulence factors analysis of TAKPN-1(K54 hvKp), NTUH-K2044 (K1 hvKp), SMU18037509 (K2 hvKp), and ATCC13883 (cKp).

With identity ≥80%, we predicted 57 virulence-related genes and 76 pathogen-related genes *via* the VFDB and the PHI database, respectively (Figure 5). Among the 57 virulence genes, 14 engaged in fimbriae synthesis, 12 were pullulanase secretion proteins, and 11 were related to iron transport and Enterobactin synthesis. Furthermore, there were five *E. coli* common pilus (ECPs) structural subunits. The 76 pathogenic factors were related to gastrointestinal infection (27/76), pneumonia (14/76), urinary tract infection (8/76), wound infection (7/76), bacteremia (7/76), and nervous system infection (3/76). We compared the bacterial virulence factors of TAKPN-1 with that of K1 hvKp, K2 hvKp, and cKp. The results showed that most virulence genes existed simultaneously in K1, K2, and K54 hvKp. However, 42 virulence genes exist in K1 or K2 hvKp, but are missing in TAKPN-1. Meanwhile, four virulence factors exist only in TAKPN-1 but not in K1 or K2 hvKp. On the other hand, 93 virulence factors exist in both cKp and hvKp, but 45 genes are missing in cKp (Figure 6).

## Discussion

The pathogen hvKp typically causes community-acquired pyogenic liver abscesses in patients with no clinical history (Arena et al., 2016). It is one of the leading cause of multisystem infection, involving pneumonia, hepatic and non-hepatic abscesses, endophthalmitis, meningitis, skin and soft tissue infections, and necrotizing fasciitis (Huang et al., 2018; Marr and Russo, 2019). Diabetes mellitus is considered as a significant risk factor for acquiring an hvKp infection, and this bacterial infection predominantly affects 55–60 year-old male individuals. In Taiwan and Singapore, hvKp was the most common cause of deep neck infections in patients with diabetes (Choby et al., 2020; Hirai et al., 2020). In the present case, the patient was a 58-year-old patient with diabetes, with similar clinical features reported previously. The clinical course of this patient revealed typical traits of hvKp infection (Eger et al., 2021). First, the infection was severe, due to the invasion of the pathogen into the bloodstream from liver abscesses. Second, multiple body sites were affected, including the respiratory and nervous systems, which is a hallmark of hvKp infection. The rapid disease progression and the severity of illness in this patient were remarkable.

So far, there are limited case reports on invasive infection caused by K54 hvKp. In order to further understand the evolutionary relationship of hvKp serotype K54, we downloaded the nucleotide sequences of eight K54 hvKp and analyzed their similarity with TAKPN-1. The results showed that seven hvKp were closely related to each other, suggesting parallel evolution of these isolates. A previous study showed that the most prevalent type found in K54 hvKp was ST29 (Turton et al., 2018). Chuang et al. (2013) reported a case of mycotic aneurysm caused by *K.pneumoniae* serotype K54 with ST29. Iwasaki et al. (2017) also reported a case of bacterial meningitis caused by hvKp capsular genotype K54 with ST 29. The latter case illustrated two significant clinical findings for cerebral focal lesion infected by K54 hvKp. First, it was agranuloma-like, similar to cryptococcoma and tuberculoma, during the subacute phase. Second, the lesion became undetectable during antibacterial therapy. Our case corroborated their clinical features. Unfortunately, we failed to isolate hvKp from CSF. The possible reasons include two aspects: first, the amount of hvKp in CSF was too small, and most bacteria invaded the brain parenchyma; second, antibiotics were used for 11 days before the CSF drainage. However, hvKp was detected in blood, liver abscess, and BALF. Therefore, it was considered that the brain lesion of this patient was caused by hvKp infection.

Except for capsule types, many other factors of hvKp attributed to its virulence and pathogenesis, including up to four siderophore systems for iron acquisition, increased capsule production, the type I and type III fimbriae, and the colibactin toxin (Wang et al., 2020). We compared the bacterial virulence factors of TAKPN-1 with that of K1 hvKp, K2 hvKp, and cKp. The results showed that three hvKp carried similar virulence factors. It is worth noting that four virulence factors exist only in TAKPN-1 but not in K1 or K2 hvKp. They are involved in iron acquisition and type VI secretion system. On the hand, 45 virulence factors only exist hvKp, which are involved in capsule production, iron acquisition and aerobactin, fimbriae related function, mucoid phenotype, and type VI secretion system. However, many virulence factors exist in both cKp and hvKp. It is possible that the different expression levels of virulence factors lead to the diversity of pathogenicity in cKps or hvKps, which needs to be further studied.

In the genome of TAKPN-1, 14 genes participated in fimbriae synthesis. Fimbriae allow bacteria to attach to the host cells to establish infection. A previous report confirmed observations that type III fimbriae contribute to biofilm formation and demonstrated that the expression of type III fimbriae is positively correlated with iron concentration (Shon et al., 2013). The invading pathogen, hvKp, encodes high affinity iron acquisition systems to counteract host nutritional immunity processes. HvKp expresses four siderophores for iron acquisition, and the accumulation of siderophore systems in hvKp suggests iron acquisition is a critical component of the emergence of this pathogen (Choby et al., 2020). Eleven genes related to iron transport and enterobactin synthesis were predicted in TAKPN-1. The definition of hvKp is controversial, however, several recent studies have reported that possessing *iutA* (aerobactin receptor-encoding gene), in addition to *rmpA*, is a characteristic hvKp trait (Hirai et al., 2020). Whole-genome sequencing showed the presence of *iutA* and absence of rmpA in TAKPN-1. Moreover, we did not find any plasmid in TAKPN-1. A previous study has also reported that some of the K54 *K. pneumoniae* of ST29 isolated from blood lack a virulence plasmid (Turton et al., 2018). We confirmed the high virulence of three clinical isolates by mouse toxicity test, which is a standard test for hvKp infection.

Although initial isolates of hvKp were antimicrobial sensitive, management challenges included rapid initiation of therapy to prevent subsequent spread, detection of occult abscess to enable source control, and appropriate site-specific therapy (for example, meningitis, endophthalmitis, and prostatic abscess; Russo and Marr, 2019). Additional imaging (including CT or MRI) may be helpful and required to recognize the infection and identify certain occult sites of infection, because site-specific antimicrobial regimens that achieve adequate drug concentrations are needed for an optimal outcome (McHardy et al., 2021). In the present case, three hvKp isolates were susceptible to all detected antibiotics, and meropenem was used because of multiple site infections, including the nervous system. This patient gradually recovered *via* antimicrobial therapy, and the abscesses shrank until disappeared. He was discharged from hospital after 55 days of antimicrobial therapy.

## Conclusion

In summary, our study described the phenotypic and molecular features of K54-ST29 hvKp causing multi-system infection in a patient with type 2 diabetes. Our study raises the concern of K54 hvKp plausibly becoming a serious health threat, considering its growing prevalence. Further epidemiological studies are warranted to elucidate the virulence factors for K54 hvKp strains, pathophysiology, and clinical features associated with their infection.

## Data Availability Statement

The datasets presented in this study can be found in online repositories. The names of the repository/repositories and accession number(s) can be found in the article/**Supplementary Material**.

## Ethics Statement

The studies involving human participants were reviewed and approved by the Ethics Committee of Taiwan City Central Hospital. The patients/participants provided their written informed consent to participate in this study. The animal study was reviewed and approved by the Ethics Committee of Taiwan City Central Hospital.

## Author Contributions

MJ and HW designed the experiments and revised the manuscript. CS carried out the experiments and wrote the manuscript. LX analyzed the data. PM contributed to experiment conception. All authors contributed to the article and approved the submitted version.

## Funding

This study was supported by the National Natural Science Foundation of China (No. 81401696) and the Shandong Provincial Natural Science Foundation of China (No. ZR2016HL44).

## Conflict of Interest

The authors declare that the research was conducted in the absence of any commercial or financial relationships that could be construed as a potential conflict of interest.

## Publisher’s Note

All claims expressed in this article are solely those of the authors and do not necessarily represent those of their affiliated organizations, or those of the publisher, the editors and the reviewers. Any product that may be evaluated in this article, or claim that may be made by its manufacturer, is not guaranteed or endorsed by the publisher.

## Supplementary Material

The Supplementary Material for this article can be found online at: https://www.frontiersin.org/articles/10.3389/fmicb.2022.872140/full#supplementary-material

Supplementary Figure S1 PFGE results for 3 hvKP isolates.

## Footnotes

^1^ www.eucast.org/clinical-breakpoint

^2^ http://www.pasteur.fr/recherche/genopole/PF8/mlst/*K.pneumoniae*.html

^3^ https://www.genome.jp/kegg/pathway.html

^4^ http://www.mgc.ac.cn/VFs/main.htm

^5^ http://www.phi-base.org/

^6^ http://www.atgc-montpellier.fr/phyml/
