# Supplementary figures and images for: Phenotypic and Molecular Characterization of K54-ST29 Hypervirulent Klebsiella pneumoniae Causing Multi-System Infection in a Patient With Diabetes
Source: Front Microbiol. 2022 May 31;13:872140. doi: 10.3389/fmicb.2022.872140 (PMC9197500; doi:10.3389/fmicb.2022.872140)

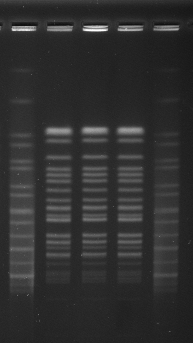

Supplement: Supplementary Figure S1 — PFGE results for 3 hvKP isolates. [file Image_1.TIF]
