# Supplementary material for: Phenotypic and Molecular Characterization of K54-ST29 Hypervirulent Klebsiella pneumoniae Causing Multi-System Infection in a Patient With Diabetes
Source: Front Microbiol. 2022 May 31;13:872140. doi: 10.3389/fmicb.2022.872140 (PMC9197500; doi:10.3389/fmicb.2022.872140)
Supplement: Supplementary file 2 [file Table_1.DOC]

Supplementary Table S1 Antibiotic susceptibilities of three *K. pneumoniae* isolates (μg/ml).

SAM, ampicillin sulbactam; TZP, piperacillin/tazobactam; ATM, aztreonam; CRO, cefatriaxone; CAZ, ceftazidime; FEP, cefepime; IPM, imipenem; MEM, meropenem; ETP, ertapenem; CN, gentamicin; AK, amikacin; CIP, ciprfloxacin; LEV, levofloxacin; STX, trimethoprim/sulfamethoxazole.

| Isolates | SAM | TZP | ATM | CRO | CAZ | FEP | IMP | MEM | ETP | CN | AK | CIP | LEV | SXT |
| --- | --- | --- | --- | --- | --- | --- | --- | --- | --- | --- | --- | --- | --- | --- |
| TAKPN-1 | <=2/1 | <=4/4 | <=1 | <=1 | <=1 | <=1 | <=0.02 | ≤0.02 | ≤0.02 | <=1 | <=2 | ≤0.25 | ≤0.25 | <=1/19 |
| TAKPN-2 | <=2/1 | <=4/4 | <=1 | <=1 | <=1 | <=1 | <=0.02 | ≤0.02 | ≤0.02 | <=1 | <=2 | ≤0.25 | ≤0.25 | <=1/19 |
| TAKPN-3 | <=2/1 | <=4/4 | <=1 | <=1 | <=1 | <=1 | <=0.02 | ≤0.02 | ≤0.02 | <=1 | <=2 | ≤0.25 | ≤0.25 | <=1/19 |
